# PoMA-10: a dual-action antiviral disrupting SARS-CoV-2 Spike–ACE2 interaction and protecting lung tissue

**DOI:** 10.3389/fphar.2026.1755268

**Published:** 2026-03-12

**Authors:** Soheun Lee, Suh Jin Yoon, Jihae Lim, Ji Hyun Oh, Jae-Sang Ryu, Gahee Kim, Hyunwoo Kang, Nayoon Jo, Sehan Lee, Sunbok Jang, Yoonji Lee, Yunjeong Park, Eun Sook Hwang

**Affiliations:** 1 College of Pharmacy and Graduate School of Pharmaceutical Sciences, Ewha Womans University, Seoul, Republic of Korea; 2 Gradutate Program in Innovative Biomaterials Convergence, Ewha Womans University, Seoul, Republic of Korea; 3 College of Pharmacy, Chung-Ang University, Seoul, Republic of Korea; 4 HITS Inc., Seoul, Republic of Korea

**Keywords:** ACE2 inhibition, acute lung injury, phenoxy-methylaniline, SARS-CoV-2, spike protein

## Abstract

This study aimed to identify small molecules that inhibit the binding of the SARS-CoV-2 Spike protein to its host receptor, angiotensin-converting enzyme 2 (ACE2), without impairing the enzymatic activity of ACE2. Such inhibitors may support the development of broad-spectrum antivirals and therapeutic strategies for emerging SARS-CoV-2 variants. Through extensive screening using both cell-free and cell-based assays, we identified phenoxy-methylaniline (PoMA) compounds as effective inhibitors of the SARS-CoV-2 Spike-ACE2 interaction. Among these, PoMA-10, featuring trifluoromethoxy and dimethylaniline moieties, exhibited the most potent inhibitory activity while preserving ACE2 enzymatic function. Computational modeling predicted direct binding of PoMA-10 to ACE2, which was corroborated by protein mobility shift assays. This was further substantiated by surface plasmon resonance analysis and molecular dynamics simulations, which confirmed the stable binding of PoMA-10 at an interface-adjacent site on ACE2 and the disruption of SARS-CoV-2 Spike–ACE2 interaction. In Vero cells, PoMA-10 significantly reduced infection by ancestral SARS-CoV-2 and the Delta and Gamma variants. Moreover, PoMA-10 alleviated lung epithelial cell damage and protected against lipopolysaccharide-induced lung injury *in vivo*. These findings demonstrate that PoMA-10 functions as a dual-action inhibitor blocking viral entry and protecting against lung injury, and highlight its potential as a therapeutic candidate in the management of COVID-19 and related pulmonary complications.

## 1 Introduction

Severe acute respiratory syndrome coronavirus 2 (SARS-CoV-2) has caused the COVID-19 pandemic, posing a major global health threat (Andersen et al., 2020; Billah et al., 2020; Narayanan et al., 2024). The high mutation rate of the virus, attributable to its low replication fidelity, has accelerated the convergence of variants (Peck and Lauring, 2018; Chen et al., 2022), thus creating a persistent threat to global health. Variants of concern, including Alpha, Beta, Gamma, Delta, and Omicron, display increased transmissibility and immune evasion, largely due to mutations in the receptor-binding domain (RBD) and N-terminal region of the Spike protein, which increase the binding affinity of Spike protein to the host cell receptor, angiotensin-converting enzyme 2 (ACE2) (Scovino et al., 2022).

Several inhibitors have been developed to primarily target viral or host enzymes. These include the antiviral drug remdesivir (RDS); the ACE2 inhibitor captopril (CAP); the protease inhibitors camostat mesylate (CM) and nafamostat mesylate (NM); and the antimalarial drug chloroquine (CQ) (Jackson et al., 2022; Wang et al., 2022; Cui et al., 2024; Dong et al., 2024). Despite the significance of viral targets, the inhibitor of virus-receptor interactions remains an attractive antiviral strategy (Hoffmann et al., 2020a; Chitsike and Duerksen-Hughes, 2021; Meganck and Baric, 2021; Shapira et al., 2022). Small molecule therapeutics, in particular, offer advantages in terms of cell permeability, metabolic stability, and cost-effectiveness (Meganck and Baric, 2021; Kronenberger et al., 2023; Yang and Wang, 2023). Yet, the continuous emergence of variants and drug resistance underscores the urgent need for broadly effective SARS-CoV-2 entry blockers (Hu et al., 2023; Iketani et al., 2023; Jochmans et al., 2023; Kiso et al., 2023; Moghadasi et al., 2023).

Diphenyl ether moieties, found in marine-derived natural products, exhibit diverse biological activities, including antiviral against viruses like influenza and herpes, as well as herbicidal properties by interfering with plant growth and metabolism (Camilleri et al., 1988; Bunyapaiboonsri et al., 2007). Inspired by the diphenyl ether scaffolds, we synthesized phenoxy-methylaniline (PoMA) derivatives—specifically CP1, CP2, and CP3—as core structures for further exploration as SARS-CoV-2 entry inhibitors. Additionally, to enhance their pharmacokinetic properties and binding affinity, we incorporated fluorine atoms, particularly focusing on trifluoromethoxy groups, which are well known recognized in medicinal chemistry for improving lipophilicity, metabolic stability, and membrane permeability (Haranahalli et al., 2019; Liu et al., 2022; Nair et al., 2022). These modifications aim to optimize the pharmacological profile of PoMA derivatives. Furthermore, we designated related compounds, CP4 and CP5, as antimicrobial peptide analogs to interfere with protein-protein interactions between SARS-CoV-2 Spike (hereafter referred to as Spike) protein and ACE2 (Park et al., 2015; Lee et al., 2024). In this study, we evaluated the effects of these PoMA derivatives, especially the fluorinated analogs, on Spike-ACE2 interactions and their potential to mitigate infection-driven inflammatory responses.

## 2 Materials and methods

### 2.1 Materials

CQ, CAP, CM, NM, RDS, MLN-4760, lipopolysaccharides (LPS), and Flag antibody were purchased from Sigma-Aldrich (St. Louis, MO, United States). Compounds in the CP series and PoMA derivatives were synthesized and purified to 98% purity by High-performance liquid chromatography(HPLC) (Lee et al., 2024). Streptavidin-allophycocyanin (APC) (405207) was purchased from BioLegend (San Diego, CA, United States). Biotinylated SARS-CoV-2 (2019-nCoV) Spike RBD-His recombinant protein (40592-V08B-B) was purchased from Sino Biological (Wayne, PA, United States).

### 2.2 ACE2: Spike inhibitor screening assays

Screening and profiling of inhibitors of the Spike–ACE2 interaction were conducted using an ACE2: Spike RBD (SARS-CoV-2) inhibitor screening assay kit (BPS Bioscience Inc., #79936, San Diego, CA, United States) according to the manufacturer’s instructions (Hoffmann et al., 2020a). In brief, a 96-well plate was coated with ACE2-His and subsequently incubated with Spike protein in the presence or absence of inhibitor compounds. The plate was washed and incubated with HRP-conjugated anti-His antibody, followed by chemiluminescence and immediate luminescence detection (Molecular Devices, Sunnyvale, CA, United States). The Spike-ACE2 blockers were additionally validated using two different inhibitor assay systems: the ACE2:Spike RBD and Spike:ACE2 binding colorimetric assay kits (RayBiotech Inc., CoV-ACE2S2 & CoV-SACE2, Peachtree Corners, GA, United States) using plate-bound ACE2 or Spike protein. Optical density was measured using a multifunctional microplate reader (Infinite 200 Pro microplate reader, Tecan, Switzerland) equipped at the Ewha Drug Development Research Core Center.

### 2.3 Cell-based Spike-hACE2 binding inhibition assay

BEAS-2B (ATCC, CRL-3588) and HEK293T (ATCC, CRL-3216) cells were maintained in DMEM with 10% FBS and transfected with pCMV3-human ACE2 (hACE2)-Flag plasmid DNA, followed by selection in the presence of hygromycin B (H772, Sigma-Aldrich). hACE2-BEAS-2B cells and hACE2-HEK293T cells expressing human ACE2 were established and subjected to the Spike binding assay. Cells (0.5 x 10^6^ cells/mL) were incubated with 100 ng of biotinylated Spike protein in the presence of dimethyl sulfoxide (1% DMSO) control, PoMA-02 (100 μM), PoMA-06 (100 μM), or PoMA-10 (100 μM) at 37 °C for 1 h. Dimethyl sulfoxide (1% DMSO) was added to the control. Cells were subsequently incubated with streptavidin-APC on ice for 20 min and analyzed using a FACS Calibur flow cytometer (BD Biosciences, San Jose, CA, United States). Cells were quantitated using the CellQuest software.

### 2.4 Molecular modeling

The X-ray crystal structure of ACE2 bound to the Spike protein (PDB ID: 6LZG) was prepared for docking by removing the Spike RBD chain and truncating all N-linked glycans (Wang et al., 2020); crystallographic water molecules were also removed. Ligands were generated and ionization states assigned at physiological pH, and induced-fit docking (IFD) was performed targeting the previously reported AlloSite3 pocket (Dutta, 2022). During IFD, residues within 5.0 Å of docked ligands were allowed to refine and side-chain optimization was carried out; 20 poses per ligand were re-docked and ranked. Predicted affinities were compared by MM-GBSA rescoring of minimized docked poses. Docking results were cross-validated against an independent ACE2 structure (PDB ID: 6M0J). Detailed procedures are provided in the Supplementary Information.

### 2.5 Molecular dynamics (MD) simulations

MD simulations started from the ACE2–Spike complex (PDB 6LZG) with the Spike placed ∼5 Å from ACE2; resolved PTMs were parameterized with AmberTools. PoMA-10 RESP charges were computed at B3LYP/6-31G** (Psi4) and GAFF2 parameters were generated via ACPYPE. Simulations were carried out using GROMACS 2024.5 with AMBER ff14SB. Systems were energy-minimized, equilibrated through six restrained stages with restraints tapered to zero, then run in production. LINCS constrained bonds to hydrogen; PME handled electrostatics; van der Waals used 0.8–1.0 nm cutoffs with dispersion corrections. Temperature was 310 K (v-rescale) and pressure 1 bar (Parrinello–Rahman). Production timestep was 2 fs; coordinates every 10 ps and energies every 2 ps. Simulations continued without regenerating velocities; COM motion removed every 100 steps. Further technical details are provided in the Supplementary Information.

### 2.6 Protein mobility shift assay

hACE2-HEK293T cells expressing Flag-tagged hACE2 were harvested and extracted with extraction buffer (50 mM HEPES, pH 7.9, 150 mM NaCl, 0.1% NP-40, 10% glycerol) supplemented with protease and phosphatase inhibitors. Protein extracts containing hACE2 were reacted with PoMA-02, PoMA-06, or PoMA-10 for 15 min at room temperature and subjected to electrophoresis on a continuous 6% native acrylamide gel for 3 h, followed by immunoblotting analysis with anti-Flag antibody. NativeMark™ unstained protein standard (Thermo Fisher Scientific, LC0725) was used for size comparison in native gel electrophoresis. The migration distance of the hACE2 complex from the wells of the acrylamide gel was measured and calculated as a percentage of the vehicle control.

### 2.7 Surface plasmon resonance (SPR) assay

SPR experiments were performed using a Nicoya OpenSPR XT rev4 (carboxyl chip) (support from NFEC-2024-09-299613). After filtering and degassing buffers, chips were activated (EDC/NHS 1:1, 100 mM each, 600 s at 20 μL/min) and ACE2 or Spike immobilized (50 μg/mL in 10 mM sodium acetate, pH 5.0) at 4 °C (∼1500 RU). Unreacted esters were blocked with 1 M ethanolamine–HCl (pH 8.5). Running buffer was PBS with 0.01% Tween-20, 0.5 mg/mL BSA, and 2% DMSO. PoMA-10 and PoMA-06 (50–200 μM) were injected at 20 μL/min; ACE2–PoMA-10 used 300 s association/dissociation, others used 300 s association and 600 s dissociation. Surfaces were regenerated with 10 mM glycine–HCl (pH 3.0, 40 s). Measurements were repeated and analyzed after reference subtraction; full details are in the Supplementary Information.

### 2.8 ACE2 enzyme activity assay

ACE2 enzyme activity was measured using a synthetic methoxycoumaryl acetyl (MCA)-based peptide substrate to release a free fluorophore. An ACE2 Inhibitor Screening Kit (ab273373, Abcam, Cambridge, United Kingdom) was used for the ACE2 enzyme activity assay. In brief, a 96-well plate was incubated with diluted ACE2 enzyme, synthetic MCA-based peptide substrate, and different concentrations of inhibitor compounds. The plate was monitored in kinetic mode for 1 h using a fluorescence microplate reader (Molecular Devices) at the Ewha Fluorescence Core Imaging Center.

### 2.9 SARS-CoV-2 infection assay

All experiments using SARS-CoV-2 were performed at Institut Pasteur Korea according to the guidelines of the Korea National Institute of Health, as reported previously (Jeon et al., 2020). In brief, Vero cells (American Type Culture Collection, CCL-81) were treated with compounds at concentrations ranging from 0.1 to 100 μM. Plates were transferred to the BSL3 containment facility and infected with SARS-CoV-2 (ancestral 0.025 MOI; Gamma 0.02 MOI; Delta 0.02 MOI; Omicron 0.045 MOI) for an additional 24 h or 48 h (for Omicron infection). Cells were incubated with anti-SARS-CoV-2 nucleocapsid antibody and Alexa Fluor 488-conjugated secondary antibody, followed by Hoechst 33342 staining. Fluorescent cells were analyzed by Operetta high-content image analysis (Perkin Elmer, Waltham, MA, United States), and the acquired images were analyzed using in-house Columbus software to quantify the cell numbers, infection ratios, and antiviral activity (Wang et al., 2022).

### 2.10 Pulmonary damage *in vitro* and *in vivo*

BEAS-2B and A549 (ATCC, CCL-185) lung epithelial cells were treated with 50 μM of amodiaquine (AQ, A2799, Sigma-Aldrich) to induce epithelial cell damage (Lee et al., 2024) and incubated with PoMA-10 (100 μM) for 24 h. Cells were harvested and subjected to reverse transcription and real-time PCR of human IL-8. For the *in vivo* assay against lung damage, 10-week-old C57BL/6 male mice were injected intraperitoneally for 5 days with DMSO (n = 6) or PoMA-10 (20 mg/kg, n = 6). Mice were anesthetized by intraperitoneal injection of Avertin and treated with LPS (5 mg/kg) by intratracheal instillation. After 48 h, mice were euthanized by CO2 inhalation using a gradual fill method (30%–70% displacement/min), followed by the collection of lung and spleen samples. All animal experiments were approved by the IACUC of Ewha Womans University (IACUC-21-072) and conducted in accordance with the international animal welfare guidelines.

### 2.11 Quantitative real-time PCR (qPCR)

Total RNA was harvested from cells and tissues and subjected to reverse transcription and qPCR using a cDNA synthesis kit and SyBr Green qPCR master mix (Thermo Fisher Scientific). Primer sets for qPCR were used as follows: 5′-agg​tgc​agt​ttt​gcc​aag-3′ and 5′-act​tct​cca​caa​ccc​tct​g-3′ for human IL-8; 5′-cat​gta​cgt​tgc​tat​cca​ggc-3′ and 5′-ctc​ctt​aat​gtc​acg​cac​gat-3′ for human actin; 5′-acc​act​tca​caa​gtc​gga​gg-3′ and 5′-tcc​agg​tag​cta​tgg​tac​tcc-3′ for mouse IL-6; 5′-tgg​acc​ttc​cag​gat​gag​gac-3′ and 5′-ttg​tcg​ttg​ctt​ggt​tct​cc-3′ for mouse IL-1β; and 5′-aga​ggg​aaa​tcg​tgc​gtg​ac-3′ and 5′-tgg​atg​cca​cag​gat​tcc-3′ for mouse actin. Relative gene expression levels were determined by normalization to actin levels.

### 2.12 H&E staining of lung tissue

Lung tissues were excised from LPS-administered mice pretreated with vehicle or PoMA-10 and sectioned at 4-μm thickness. Tissue sections were stained with an H&E staining kit (Abcam, AB245880) and then examined under a light microscope (Nikon Eclipse Ci, Nikon, Japan).

### 2.13 Statistical analysis

All experiments were performed independently at least five times, and the data are expressed as the mean ± standard error of the mean (SEM). Statistical significance was determined using a two-tailed Student’s t-test for comparisons between two groups, or one-way analysis of variance (ANOVA) followed by Tukey’s HSD *post hoc* test for multiple comparisons. A P value of <0.05 were considered statistically significant.

## 3 Results

### 3.1 Identification of Spike–ACE2 binding inhibitors among small molecules

**FIGURE 1:**
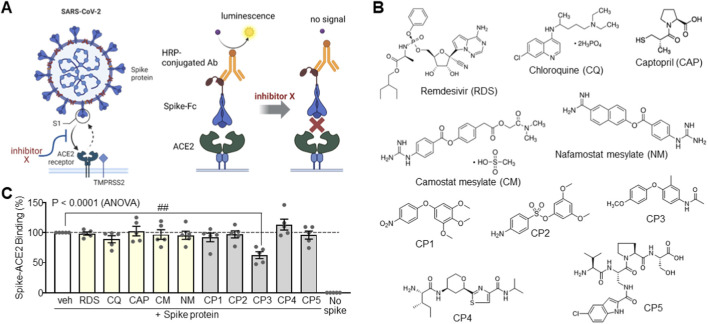
Screening of Spike-ACE2 interaction blockers. **(A)** Assay system for screening the Spike-ACE2 interaction inhibitors using chemiluminescence detection. **(B)** Chemical structures of antiviral agents and synthetic compounds. **(C)** Inhibitory effects of compounds on Spike-ACE2 interaction. DMSO was used as a vehicle (veh) control. Data represent the mean ± SEM of five independent experiments. ##, P < 0.005 by ANOVA with Tukey’s HSD *post hoc* test.

To discover small molecules capable of inhibiting SARS-CoV-2 entry by disturbing the Spike-ACE2 interaction, we performed an inhibition assay using a luminescence-based Spike-ACE2 binding model. Luminescence increases upon Spike-ACE2 binding and decreases when binding is blocked, providing a robust platform for screening (Figure 1A). We tested antiviral agents (RDS and CQ), membrane receptor inhibitors (CAP, CM, and NM) (Hoffmann et al., 2021; Hoffmann et al., 2020c; Gao et al., 2021), and various diphenyl ether analogs (Figure 1B). As expected, RDS and CQ, which act on viral replication (Hoffmann et al., 2020b), did not interfere directly with Spike-ACE2 binding. CAP, CM, and NM, which target viral proteins, failed to affect the interaction. In contrast, screening of diphenyl ether derivatives (CP1-CP3) and functional peptides (CP4 and CP5) identified CP3 as a potent inhibitor of Spike-ACE2 binding (Figure 1C).

### 3.2 Structural optimization of PoMA-01 for enhanced Spike-ACE2 binding inhibition

**FIGURE 2:**
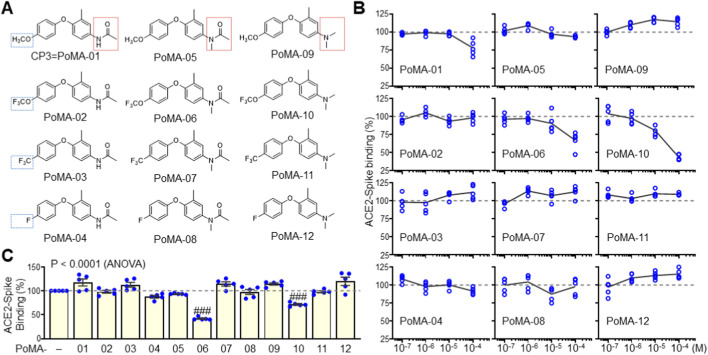
Inhibition activity of PoMA derivatives on Spike-ACE2 interaction. **(A)** Chemical structures of synthetic PoMA derivatives. **(B)** Inhibition assay of PoMA derivatives for Spike-ACE2 interaction using chemiluminescence detection. The inhibitory activity of PoMA derivatives on Spike-ACE2 interaction was assayed in triplicate at various concentrations and repeated five times independently. Data are expressed as the mean ± SEM by calculating as a percentage compared to the mean value of the vehicle-treated control of five independent experiments. **(C)** Inhibition assay of 100 μM PoMA derivatives on Spike-ACE2 binding using soluble Spike RBD and plate-bound ACE2. The effect of PoMA derivatives on Spike binding to plate-bound ACE2 was expressed as a percentage compared to vehicle control. Data are the mean ± SEM of five independent experiments. ###, P < 0.0005 by ANOVA with Tukey’s HSD *post hoc* test.

To enhance inhibitory efficacy, we performed rational structural optimization of CP3, hereafter referred to as phenoxy-methylaniline-01 (PoMA-01). Building on the diphenyl ether scaffold, we introduced modifications, including trifluoromethoxy, trifluoromethyl, and fluorine substitutions on the left aromatic ring, and methylation of the amine group on the right aromatic ring (Figure 2A) (Lee et al., 2024). Compounds bearing trifluoromethoxy, trifluoromethyl, or fluorine alone did not significantly inhibit binding. However, PoMA-06, which contains a trifluoromethoxy group and an *N*-methylacetamide, exhibited enhanced inhibition. Further modification yielded PoMA-10, in which the acetyl group was replaced with a methyl group. PoMA-10 demonstrated superior inhibition of Spike–ACE2 binding (Figure 2B), underscoring the importance of both the trifluoromethoxy and dimethyl substitutions. These findings were confirmed using the ACE2:Spike RBD (SARS-CoV-2) inhibitor screening assay, where PoMA-06 and PoMA-10 both significantly reduced the binding of the Spike RBD to plate-bound ACE2 (Figure 2C).

### 3.3 Inhibition of spike binding to ACE2-expressing cells

**FIGURE 3:**
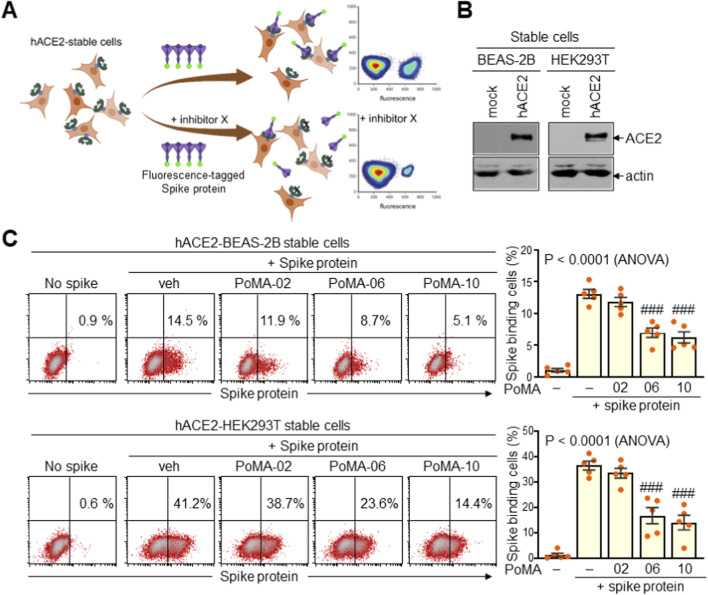
Validation of Spike-ACE2 binding inhibition of PoMA derivatives in cell-based assay. **(A)** Schematic diagram of the cell-based inhibition assay system. **(B)** Expression of ACE2 in stable BEAS-2B and HEK293T cells expressing ACE2. Representative images from five experiments are shown. **(C)** Cell-based inhibition assay. Stable hACE2-BEAS-2B and hACE2-HEK293T cells were incubated with fluorescence-tagged Spike protein in the presence of vehicle (veh) or PoMA derivatives and subjected to flow cytometry and quantitative analysis. Representative images of five independent experiments are presented, and data are given as the mean ± SEM (n = 5). ###, P < 0.0005 by ANOVA with Tukey’s HSD *post hoc* test.

We next evaluated whether PoMA-06 and PoMA-10 could inhibit Spike binding to ACE2 on the cell surface. Stable ACE2-expressing human cell lines were established in normal lung epithelial BEAS-2B and transformed HEK293 via transfection with human ACE2, with high expression confirmed by immunoblotting (Figures 3A,B). Cells were treated with PoMA-02, PoMA-06, and PoMA-10—all containing a trifluoromethoxy group—and binding of fluorescently tagged Spike protein was assessed by flow cytometry. While PoMA-02 showed no effect, PoMA-06 and PoMA-10 significantly reduced Spike binding to ACE2-expressing cells, with PoMA-10 exhibiting stronger inhibition (Figure 3C). These results confirm that PoMA-10 effectively disrupts Spike protein binding to ACE2 on the cell membrane.

### 3.4 Predicted binding site of PoMA compounds on ACE2

**FIGURE 4:**
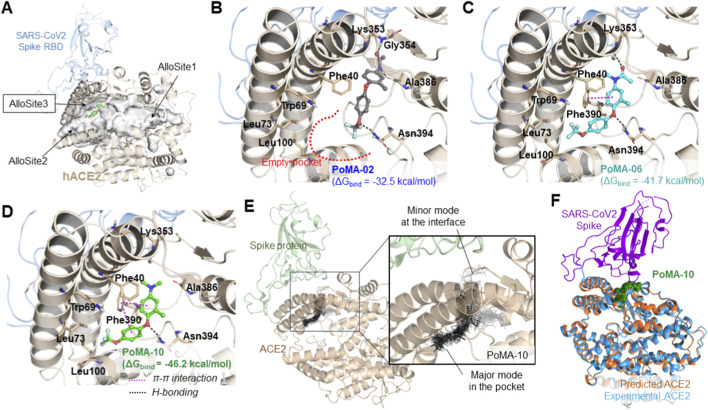
Structural modeling of PoMA derivatives binding to ACE2. **(A)** Structure of human ACE2 bound to the RBD of SARS-CoV-2 and possible binding sites for PoMA derivatives binding: AlloSite1, AlloSite2, and AlloSite3 in the ACE2 cavity. The binding of PoMA-10 (green ball-and-stick structure shown) to AlloSite3, located close to the binding site with the Spike RBD in ACE2, was predicted. **(B–D)** Predicted binding models of PoMA-02 **(B)**, PoMA-06 **(C)**, and PoMA-10 **(D)** in AlloSite3 based on IFD simulation. The interacting residues are shown on the sticks and labeled. Hydrogen bonds and π–π interactions are marked with black and magenta dashed lines, respectively. In **(B)**, the red dashed line denotes a space in the binding mode of PoMA-02. **(E)** Overlaid multiple docking modes of PoMA-10 near the Spike-ACE2 interface. Black sticks represent major conformations, and gray sticks indicate minor conformations observed in the docking runs. **(F)** Structural alignment of the predicted ACE2–PoMA-10 complex with the experimentally resolved Spike-ACE2 complex. The predicted and experimental ACE2 structure is shown in orange and blue, respectively. The SARS-CoV-2 Spike RBD is depicted in violet and PoMA-10 is represented in green.

We then examined whether PoMA derivatives directly interact with ACE2 and modulate the Spike-ACE2 complex. AlloFinder and CAVER analyses (Dutta, 2022) identified three allosteric sites in ACE2, of which AlloSite3—adjacent to the Spike-binding interface—was the most favorable (Baig et al., 2020; Mackin et al., 2022). IFD simulations predicted stable binding of PoMA-06 and PoMA-10 at AlloSite3, whereas PoMA-02 showed unstable interactions and failed to bind persistently (Figure 4A,B; Supplementary Figure S1–S3). PoMA-06 formed stable π–π interactions with F390 and hydrogen bonds with K353 and N394, and PoMA-10 formed additional interactions, including its trifluoromethoxy group with L100 (Figure 4C,D). Prime MM-GBSA rescoring (OPLS4/VSGB) yielded ΔG_bind_ values of −46.2, −41.7, and −32.5 kcal·^−1^ for PoMA-10, PoMA-06, and PoMA-02, respectively, supporting PoMA-10 as the strongest binder at AlloSite3. MD simulations confirmed that the ACE2–PoMA-10 complex retained a confirmation close to apo ACE2 (Cα RMSD <2 Å) (Figure 4E,F). Over 300 ns, the Spike protein progressively engaged apo ACE2 with inter-residue distances falling below 3.5 Å, whereas PoMA-10 increased interfacial fluctuations and reduced residue-level contacts (<3.5 Å) between Spike and ACE2 (Supplementary Videos S1, S2; Supplementary Figure S4–S6). These data indicate that PoMA-10 disrupts stable Spike-ACE2 binding via interface-adjacent modulation, likely through steric or electrostatic effects.

### 3.5 Direct binding of PoMA-10 to ACE2 without affecting enzymatic activity

**FIGURE 5:**
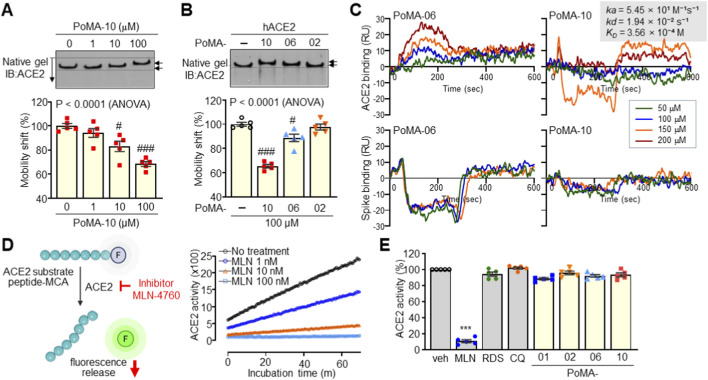
Binding of PoMA-10 to ACE2 without affecting ACE2 enzyme activity. **(A)** Mobility shift assay of ACE2 by binding with PoMA-10. ACE2 protein was incubated with different concentrations of PoMA-10 and resolved by native gel electrophoresis, followed by immunoblotting (IB) with ACE2 antibody and quantitative analysis. The mobility of PoMA-10-bound ACE2 was calculated by comparing it with the mobility of free ACE2. **(B)** Mobility shift assay of ACE2 in the presence of 100 μM of PoMA-02, PoMA-06, and PoMA-10. ACE2 mobility shift in the presence of compounds was expressed as a percentage of free ACE2 mobility. Representative images of five independent experiments are presented in A and B, and data are given as the mean ± SEM (n = 5). #, P < 0.05; ###, P < 0.0005 by ANOVA with Tukey’s HSD *post hoc* test. **(C)** SPR assay of PoMA compounds for binding to ACE2 and Spike protein, using a Biacore T200. Multiple concentrations of the compounds were injected into the equipment that contains an ACE2-bound CM5 chip. Binding values are expressed as response units (RU). All assays were performed in triplicate. **(D)** ACE2 enzyme activity assay using synthetic MCA-based peptide substrate. Suppression of ACE2 enzyme activity by different concentrations of MLN-4760 (1, 10, and 100 nM) was repeated five times in triplicate and representative results are shown. **(E)** Effects of RDS, CQ, and PoMA derivatives on ACE2 enzyme activity. ACE2 enzyme activity was determined in the presence of MLN-4760 (100 nM), RDS (100 μM), CQ (100 μM), or PoMA derivatives (100 μM) and expressed as a percentage relative to the vehicle (Veh) control. Five independent assays were performed. ***P < 0.0005 by two-tailed Student’s t-test.

We then validated direct binding of PoMA-10 to ACE2 using native polyacrylamide gel electrophoresis. PoMA-10 treatment induced a significant mobility shift of intracellular ACE2 protein at a concentration of 100 μM (Figure 5A). PoMA-02 had no significant effect, whereas PoMA-06 slightly delayed ACE2 protein mobility (Figure 5B). Further SPR analyses demonstrated that PoMA-06 and PoMA-10 exhibited negligible binding affinity for the Spike protein, whereas they showed distinct interaction profiles with the ACE2 protein (Figure 5C). PoMA-06 displayed a concentration-dependent association with ACE2 but failed to reach a clear saturation point, suggesting a relatively weak or transient binding mode. In contrast, PoMA-10 demonstrated a robust dose-dependent association with ACE2. Kinetic analysis of the PoMA-10/ACE2 interaction revealed an association rate constant (*ka*) of 5.45 × 10 M^–1^s^–1^ and a dissociation rate constant (*kd*) of 1.94 × 10^−2^ s^–1^. The resulting equilibrium dissociation constant (*K*_*D*_) was calculated to be 3.56 × 10^−4^ M (356 µM). While the binding affinity falls within the high micromolar range—a characteristic frequently observed in low-molecular-weight allosteric modulators—the sensorgrams clearly demonstrate specific, reproducible target engagement. We also confirmed that the known ACE2 inhibitor, MLN-4760, suppressed ACE2 enzymatic activity at the nanomolar concentration. PoMA-10, RDS, and CQ did not affect ACE2 activity, even at a high concentration of 100 μM (Figures 5D,E). These findings demonstrate that PoMA-10 directly binds ACE2 without compromising its enzymatic function.

### 3.6 Inhibition of SARS-CoV-2 infection in vero cells by PoMA-10

**FIGURE 6:**
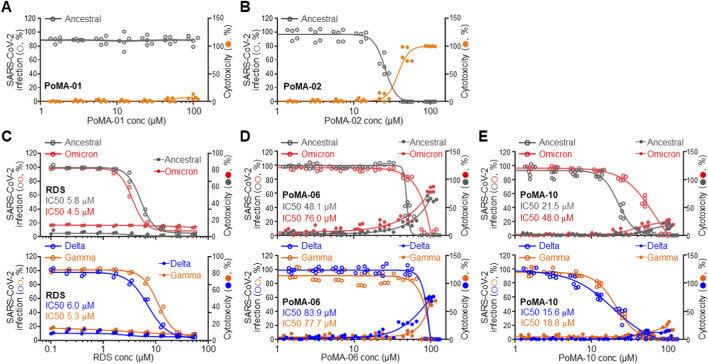
Inhibition of SARS-CoV-2 infection rate by PoMA-10. **(A and B)** Effects of PoMA-01 **(A)** and PoMA-02 **(B)** on the infection rate (open gray circles) of ancestral SARS-CoV-2 in Vero cells, and their cytotoxicity (closed orange circles) in virus-infected Vero cells. **(C–E)** Dose-response curves showing infection inhibition and cytotoxicity by RDS (n = 2, **(C)**, PoMA-06 (n = 5, **(D)** and PoMA-10 (n = 5, **(E)**. Left y-axis: infection rate (open circles) for ancestral SARS-CoV-2 (gray) and its Delta (blue), Gamma (orange), and Omicron (red) variants in Vero cells. Right y-axis: cytotoxicity in virus-infected Vero cells (closed circles). Data are expressed as the mean ± SEM (n = 2–5). IC50 values were derived by a sigmoidal dose–response curve using GraphPad Prism software.

To assess the antiviral activity of PoMA-10 against SARS-CoV-2 infection, we measured the infection rates of the ancestral SARS-CoV-2 and major variants in Vero cells. PoMA-01 had no effect on cell viability or infection, whereas PoMA-02 reduced infection only at cytotoxic concentration (Figure 6A,B). The antiviral RDS, used as a positive control, dose-dependently reduced the infection rates of the ancestral SARS-CoV-2 and major variants, with IC50 values below 10 μM (Figure 6C). PoMA-06 showed weak antiviral activity against the ancestral SARS-CoV-2 and major variants with IC50 values above 50 μM, along with cytotoxicity (Figure 6D). In contrast, PoMA-10 exhibited low cytotoxicity and potent inhibition (IC50 = 21.5 μM) against the ancestral virus, retaining significant activity against Delta and Gamma, and moderate potency against Omicron (Figure 6E). These results suggest the potential of PoMA-10 as an antiviral agent against infectious disease caused by diverse SARS-CoV-2 variants.

### 3.7 Protective effects of PoMA-10 against lung injury

**FIGURE 7:**
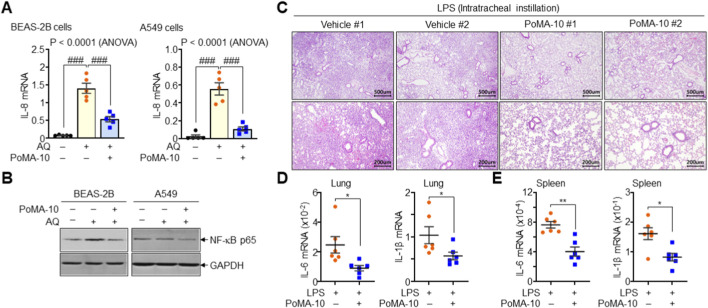
Inhibition of lung epithelial cell damage and acute lung injury by PoMA-10. **(A)** Effects of PoMA-10 on IL-8 expression induced by AQ (50 μM) in BEAS-2B and A549 lung epithelial cells. Data are expressed as the mean ± SEM (n = 5). ###, P < 0.0001, by ANOVA with Tukey’s HSD *post hoc* test. **(B)** Effects of PoMA-10 on NF-κB expression in BEAS-2B and A549 cells. Representative images of three independent experiments are presented. **(C–E)** Protective effects of PoMA-10 against LPS-induced acute lung injury. Mice were injected with either vehicle (Veh, n = 6) or PoMA-10 (20 mg/kg, n = 6) for 5 consecutive days and injected with LPS (5 mg/kg) through intratracheal instillation. Lung tissue sections were subjected to H&E staining **(C)**. Total RNA was isolated from lung **(D)** and spleen **(E)** to analyze the pro-inflammatory cytokines IL-6 and IL-1β. Data are expressed as the mean ± SEM (n = 6). *P < 0.05 and **P < 0.005 by two-tailed Student’s t-test.

Given its structural similarity to MPoMA, a known anti-inflammatory compound (Lee et al., 2024), we investigated whether PoMA-10 affects IL-8 expression in BEAS-2B and A549 lung epithelial cells damaged by high concentrations of amodiaquine (AQ). In BEAS-2B and A549 cells, AQ significantly increased IL-8 expression, a response that was markedly reduced by PoMA-10 (Figure 7A). Furthermore, PoMA-10 suppressed the expression of the inflammatory mediator NF-κB (Figure 7B), collectively indicating that PoMA-10 exerts both anti-inflammatory activity and cytoprotective effects on lung epithelial cells. In an LPS-induced mouse model of acute lung injury, intraperitoneal injection of PoMA-10 markedly reduced immune cell infiltration and tissue damage (Figure 7C). Levels of the pro-inflammatory cytokines IL-6 and IL-1β in the lung and spleen were also significantly decreased by PoMA-10 treatment (Figures 7D,E). These results definitively show that PoMA-10 has significant potential as a therapeutic agent against both virus-induced and inflammation-induced lung damage.

## 4 Discussion

In this study, we identified PoMA-10 as a lead small molecule that blocks Spike-ACE2 interaction, while exerting potent anti-inflammatory effects. Structural optimization revealed that incorporating a trifluoromethoxy group and an *N,N*-dimethyl substitution were critical to enhancing viral entry inhibition against SARS-CoV-2 and its major variants.

PoMA-10 is derived from a marine-inspired diphenyl ether scaffold (Camilleri et al., 1988; Bunyapaiboonsri et al., 2007). While our previous study on MPoMA demonstrated cytoprotective effects but limited viral entry inhibition (Lee et al., 2024), the structural transition to a trifluoromethoxy group in PoMA-10 proved essential to bridging this gap, successfully achieving both potent antiviral and anti-inflammatory activities.

To validate the direct engagement of PoMA-10 with the host receptor, we performed SPR analysis, which yielded a calculated *K*_*D*_ of 356 µM. While this affinity is moderate, it aligns with the micromolar IC50 values observed in our functional assays. The efficacy of PoMA-10, despite this moderate affinity, is hallmark of interface-adjacent allosteric inhibitors. In such cases, biological activity arises from inducing specific conformational constraints that disrupt protein–protein interactions (PPI) rather than requiring high-affinity occupancy of a primary binding pocket. By modulating ACE2 accessibility to the Spike protein in this manner, PoMA-10 provides a resilient therapeutic strategy that is less susceptible to viral mutations (Awad et al., 2023).

Although the micromolar potency of PoMA-10 is higher than that of direct-acting antivirals like RDS, its clinical value lies in its high genetic barrier to resistance and dual-action capability. The experimental use of 100 µM *in vitro* was intended to delineate maximal inhibitory potential. Crucially our *in vivo* toxicity data (Supplementary Figure S7)—which showed no adverse effects at doses up to 1,000 mg/kg—suggests a remarkably wide therapeutic window. This safety margin supports the feasibility of localized delivery, such as inhalation or intranasal administration, to achieve effective concentrations at the primary respiratory infection site while minimizing systemic exposure.

To ensure clinical relevance, beyond the innate immune-deficient Vero cell model, we validated PoMA-10 in human-derived immunocompetent lines (BEAS-2B and HEK293T), confirming its efficacy in a context mimicking the human respiratory environment. Molecular modeling suggests that PoMA-10 binds to an interface-adjacent site near the Spike-binding interface without affecting ACE2 enzymatic activity, unlike ACE2 inhibitor MLN-4760 (Berenyiova et al., 2021; Nami et al., 2021; Wang et al., 2024). This allosteric modulation is a key advantage; by inducing subtle conformational shifts at the PPI interface rather than blocking the active site, PoMA-10 prevents viral entry without interfering with the essential physiological functions of ACE2.

Notably, our data suggest that PoMA-10’s antiviral and anti-inflammatory activities operate via independent, parallel mechanisms rather than a single causal chain. While a reduction in viral entry naturally leads to decreased pathogen-induced inflammation, the efficacy of PoMA-10 in the sterile LPS-induced lung injury model—where no viral replication occurs—demonstrates a direct, intrinsic anti-inflammatory capacity. This dual-target profile allows PoMA-10 to block extracellular viral entry via ACE2 modulation while simultaneously—likely through cell permeation—inhibiting intracellular NF-κB-mediated signaling. This ensures that the inflammatory cascade can be mitigated even if some viral particles bypass the initial entry barrier.

Despite these promising findings, further studies on pharmacokinetics and efficacy in SARS-CoV-2-specific models (e.g., K18-hACE2 mice) remain necessary (Yinda et al., 2021; Zheng et al., 2021). Advanced MD-based network analyses, including dynamic cross-correlation matrix and principal component analysis, are planned to further refine our understanding of PoMA-10’s host-modulatory mechanisms. Overall, PoMA-10 exhibits a multifunctional profile as a next-generation host-targeted antiviral with robust dual-action benefits.

## 5 Conclusion

PoMA-10 was identified as a potent small-molecule inhibitor of SARS-CoV-2 entry. By binding directly to an allosteric site on ACE2, it effectively blocked viral entry across multiple variants without compromising the receptor’s enzymatic activity. Beyond its antiviral effect, PoMA-10 mitigated inflammatory lung damage, highlighting its potential as a dual-action therapeutic strategy.

## Data Availability

The original contributions presented in the study are included in the article/Supplementary Material, further inquiries can be directed to the corresponding authors.
