# Supplementary material for: PoMA-10: a dual-action antiviral disrupting SARS-CoV-2 Spike–ACE2 interaction and protecting lung tissue
Source: Front Pharmacol. 2026 Mar 12;17:1755268. doi: 10.3389/fphar.2026.1755268 (PMC13017958; doi:10.3389/fphar.2026.1755268)
Supplement: Supplementary file 1 [file DataSheet1.pdf]

PoMA-10: A Dual-Action Antiviral Targeting Spike-ACE2 Interaction with Antiviral and Lung-Protective Efficacy

Soheun Lee[1,5], Suh Jin Yoon[1,2,5], Jihae Lim[1], Ji Hyun Oh[1], Jae-Sang Ryu[1], Gahee Kim[3], Hyunwoo Kang[4], Nayoon Jo[1,2], Sehan Lee[4], Sunbok Jang[1,2], Yoonji Lee[3], Yunjeong Park[1,*], and Eun Sook Hwang[1,2,*]

Supplementary information

This file includes detailed methods, supplementary figures S1–S6, supplementary videos 1–2, and references.

Supplementary methods

SI.1. Molecular modeling

The X-ray crystal structure of ACE2 bound to the SARS-CoV-2 spike protein (PDB ID: 6LZG) was obtained from the Protein Data Bank [1] and prepared using the Protein Preparation Wizard in Schrödinger (2023-4 release). For docking, the spike RBD chain was removed and all resolved N-linked glycans were truncated to maintain a consistent representation of the ACE2 surface; all crystallographic water molecules were removed. Bond orders, charges and protonation states were assigned, hydrogen atoms were added, and the structure was minimized to a root-mean-square deviation (RMSD) of 0.30 Å. The catalytic Zn^{2+} ion and its coordinating residues (H374, H378, E402) were retained in their native protonation states to preserve the tetrahedral coordination geometry. Ligands were generated using the *LigPrep* module with the OPLS4 force field and ionization states were assigned with *Epik* at pH 7.4.

Induced-fit docking (IFD) was carried out with extended sampling using a grid box centered on the previously reported AlloSite3 pocket (residues F356, D355, D382, G354, D350, L351, Y385, A386, G352, K353, R393, F390, F40, E37) [2]; the grid center coordinates were (−30.64, 23.84, −9.09) with inner and outer box dimensions of 10 Å and 30 Å, respectively. During docking refinement, residues within 5.0 Å of the ligands were allowed to move and side-chain optimization was performed using Prime. Twenty poses per ligand were re-docked and ranked by Glide SP. Cross-docking validation was performed using an independent ACE2 complex (PDB ID: 6M0J), and PoMA-10 exhibited similar binding poses in both templates (heavy-atom RMSD ≈ 1.5 Å), supporting the structural robustness of the identified interface-adjacent binding site (AlloSite3).

To quantitatively compare predicted affinities of the key molecules, Molecular Mechanics, General Born Surface Area (MM-GBSA) calculations were performed. Binding free energies (ΔG_{bind}) of PoMA analogs were predicted using the Prime MM-GBSA module implemented in Schrödinger. Docked poses obtained from Glide were locally minimized prior to MM-GBSA rescoring. The calculations employed the OPLS4 force field and the Variable-Solvent Generalized Born (VSGB) implicit solvation model, with protein flexibility allowed for residues within 7 Å of each ligand. The hierarchical sampling method was applied to efficiently explore local conformational space. The binding free energy was computed as: $\Delta G_{bind} = G_{complex} -$

($G_{protein}$ + G_{ligand}), where G represents the minimized free energy of each component. All molecular graphics were prepared with PyMOL v.2.5.2.

SI.2. Molecular Dynamics (MD) simulation setup

To study ACE2-Spike interaction, systems were built from PDB 6LZG. Two cases were simulated: (i) ACE-Spike (apo) and (ii) ACE2–Spike with PoMA-10 bound to ACE2. N-linked glycans and other post-translational modifications (PTMs) present in 6LZG were parameterized using AmberTools. PoMA-10 was parameterized via ACPYPE [3] to generate gaff2-compatible parameters (Generate AMBER force field 2) [4]; partial atomic charges were computed using the Psi4 Python API at the DFT/B3LYP-6-31G** level with the RESP protocol. The initial center-of-mass (COM) separation between ACE2 and Spike was 5.44 Å.

All MD simulations used GROMACS 2024.5 with the AMBER ff14SB protein force field. Zn^{2+} parameters compatible with ff14SB were adopted [5]. Systems were solvated with TIP3P water and neutralized to 150 mM NaCl.

Following energy minimization and NVT/NPT equilibration, six restrained equilibration steps (step4.1 to step4.6) were performed before the production run. Positional and dihedral restraints were reduced progressively (4200, 1680, 840, 420, and 0 kJ mol^{-1} nm^{-2} for each step; CHARMM-GUI position restraint style [6]).

Each equilibration stage employed the Verlet cutoff scheme, and all bonds involving hydrogen atoms were constrained using the LINCS algorithm. Integration timesteps ranged from 1–2 fs, and individual equilibration durations varied between 1.25 and 5 ns. Coordinates were saved every 5 ps. Periodic boundary conditions were applied (pbc = xyz), and reference coordinate scaling was performed by center of mass (refcoord_scaling = com).

Production simulations were performed with a 2 fs timestep. Total stimulated times reported include 100 ns for protein-protein interaction analyses (2 fs × 5 × 10^6 steps; complex 40 ns, protein-protein 100 ns), unless otherwise stated. Coordinates were saved every 10 ps; energies and log data were written every 2 ps. Center-of-mass motion was removed every 100 steps. Simulations were continued without regenerating velocities (gen_vel = no, continuation = yes).

Electrostatic interactions were calculated using the Particle Mesh Ewald (PME) method with a real-space cutoff of 0.8 nm abd rlist = 1.2 nm, while van der Waals interactions were treated with a 0.8–1.0 nm scheme using a potential-shift modifier. Dispersion corrections were applied to both energy and pressure (DispCorr = EnerPres). Temperature was maintained at 310 K using the velocity-rescaling (v-rescale) thermostat; in later equilibration stages, solute and solvent groups were coupled separately to maintain thermal stability. Pressure was controlled isotropically with the Parrinello–Rahman barostat (τp = 2.0 ps). All bonds were constrained using the LINCS algorithm (order = 4, iteration = 1).

For analysis and reproducibility, trajectory metrics (COM distance versus time, interfacial contact counts, ΔSASA, and interaction-energy time series) were computed from production data.

SI.3. Surface plasmon resonance (SPR) assay

SPR experiments were performed on a Nicoya OpenSPR XT rev4 (Nicoya Lifesciences) using a carboxyl-functionalized sensor chip. All buffers and samples were filtered (0.22 μm) and degassed before use. Chip activation and ligand immobilization were performed at 4 °C to preserve ligand integrity; analyte injections and regeneration steps were carried out at room temperature.

The sensor surface was activated by injecting a 1:1 mixture of 100 mM N-(3-dimethylaminopropyl)-N′-ethylcarbodiimide hydrochloride (EDC) and 100 mM N-hydroxysuccinimide (NHS) for 600 s at 20 μL/min. Human ACE2 and SARS-CoV-2 Spike proteins (ligands) were prepared to 50 μg/mL in 10 mM sodium acetate buffer (pH 5.0) and immobilized on separate flow channels via standard amine coupling at 10 μL/min. Association and dissociation phases during immobilization were 600 s and 300 s, respectively, yielding ~1,500 response units (RU) for each ligand. Remaining active esters were blocked with 1 M ethanolamine–HCl (pH 8.5) for 300 s at 20 μL/min, followed by a 300 s dissociation.

The running buffer was PBS contianing 0.01% Tween-20, 0.5 mg/mL bovine serum albumin (BSA), and 2% dimethyl sulfoxide (DMSO). Reference channels underwent identical activation and blocking but no ligand immobilization; all sensorgrams were reference-subtracted.

PoMA-10 and PoMA-06 (analytes) were diluted in running buffer to 50, 100, 150, and 200 μM and injected at 20 μL/min over ACE2 or Spike immobilized channels. For ACE2–PoMA10, where clear binding was observed, association and dissociation phases were 300 s each. For other ligand-analyte combinations, an association of 300 s and an extended dissociation of 600 s were used to better assess weak or transient binding. Surfaces were regenerated after each injection with a 40 s pulse of 10 mM glycine–HCl (pH 3.0). All injections were performed in duplicate. Sensorgrams were reference-subtracted and processed to remove bulk refractive index effects before analysis. after subtraction of the reference channel signal.

Supplementary Figures and Videos

Figure S1. Molecular docking of PoMA compounds to allosteric site 1 of ACE2

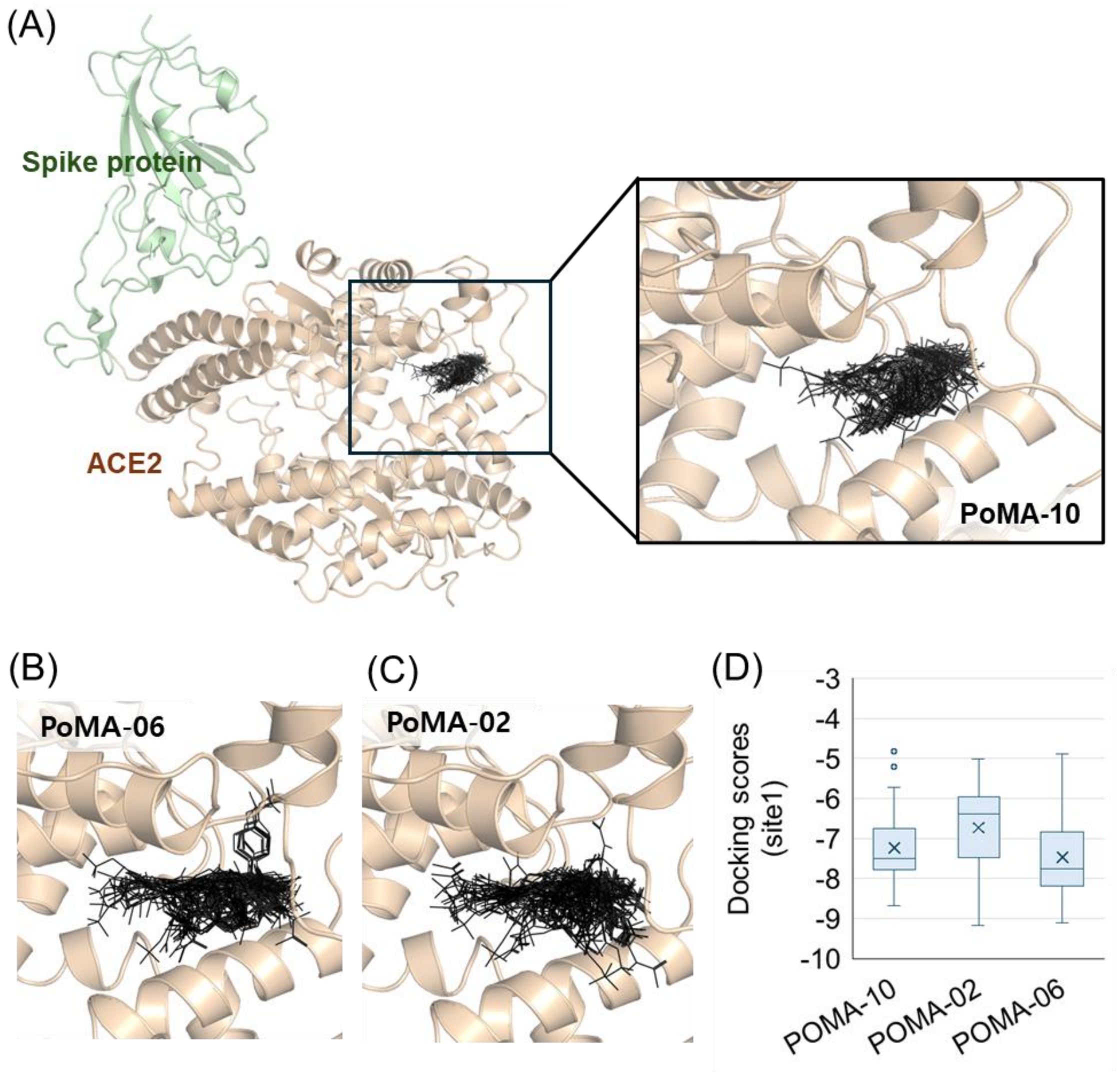

Figure S1. **Docked conformations of PoMA compounds at allosteric site 1 of ACE2.** (**A**–**C**) Overlay of multiple docking poses for PoMA-10, PoMA-06, and PoMA-02, respectively, at site 1. Docking simulations show that all three compounds adopt highly variable and inconsistent binding modes at this site, indicating unstable or non-specific interactions. (**D**) Distribution of docking scores (binding affinities in kcal/mol) for each compound at site 1. Boxes indicate interquartile ranges (25–75%), horizontal lines denote median scores, and whiskers represent the full range of values. Despite negative docking scores suggesting nominal affinity, the absence of reproducible binding poses suggests that site 1 is unlikely to support reliable ligand binding.

Figure S2. Molecular docking of PoMA compounds to allosteric site 2 of ACE2

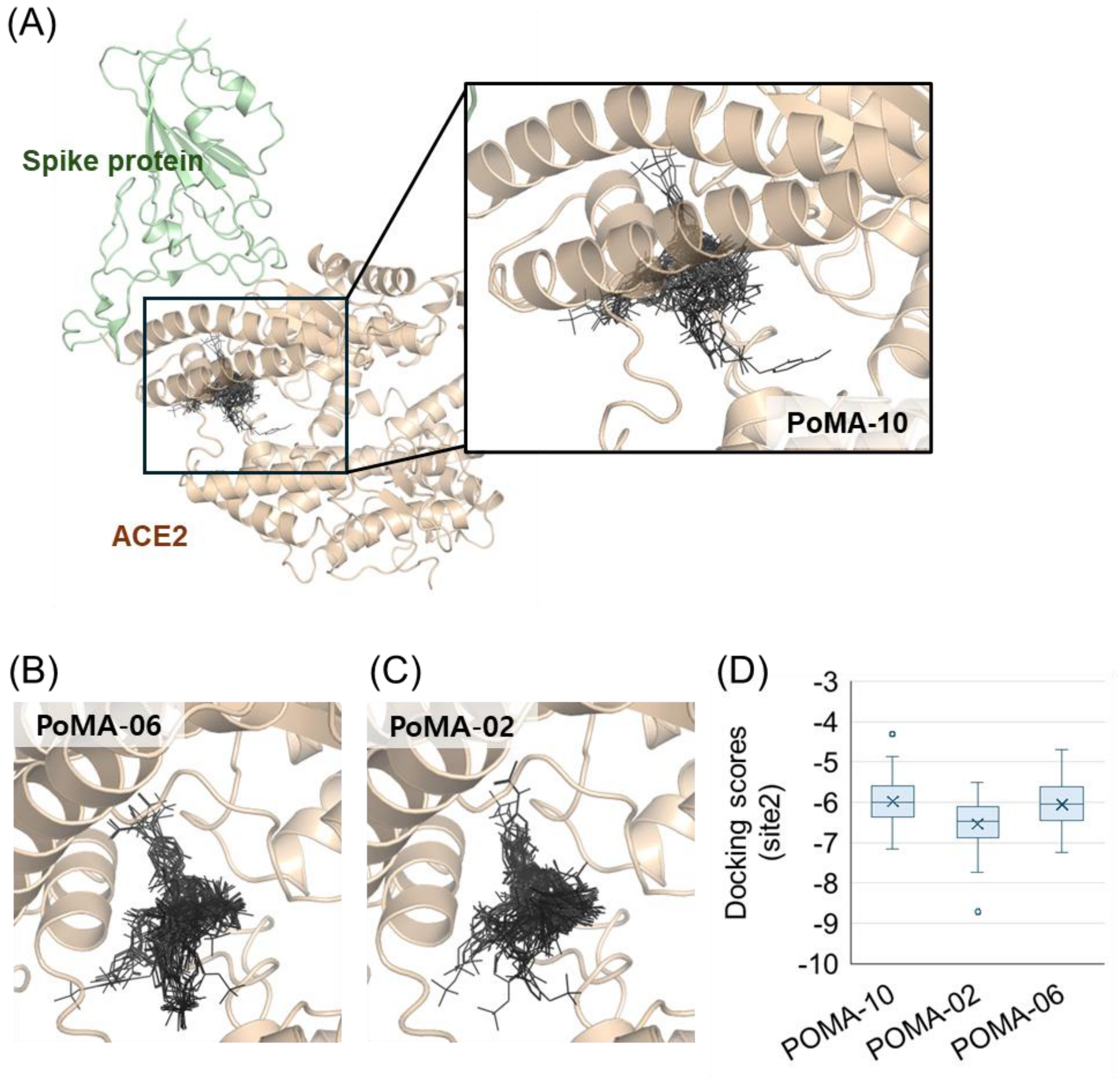

Figure S2. **Docked conformations of PoMA compounds at allosteric site 2 of ACE2.** (**A**–**C**) Overlays of multiple docking poses for PoMA-10, PoMA-06, and PoMA-02, respectively, at site 2. The docking simulations show that all three compounds adopt variable and inconsistent binding modes at this site, indicating unstable or unreliable interactions. (D) Distribution of docking scores (binding affinity values, kcal/mol) for each compound at Site 2. Although the scores are nominally favorable (negative values), the absence of reproducible binding poses across simulations suggests that site 2 is unlikely to be a reliable binding site for these compounds.

Figure S3. Molecular docking of PoMA compounds to allosteric site 3 of ACE2

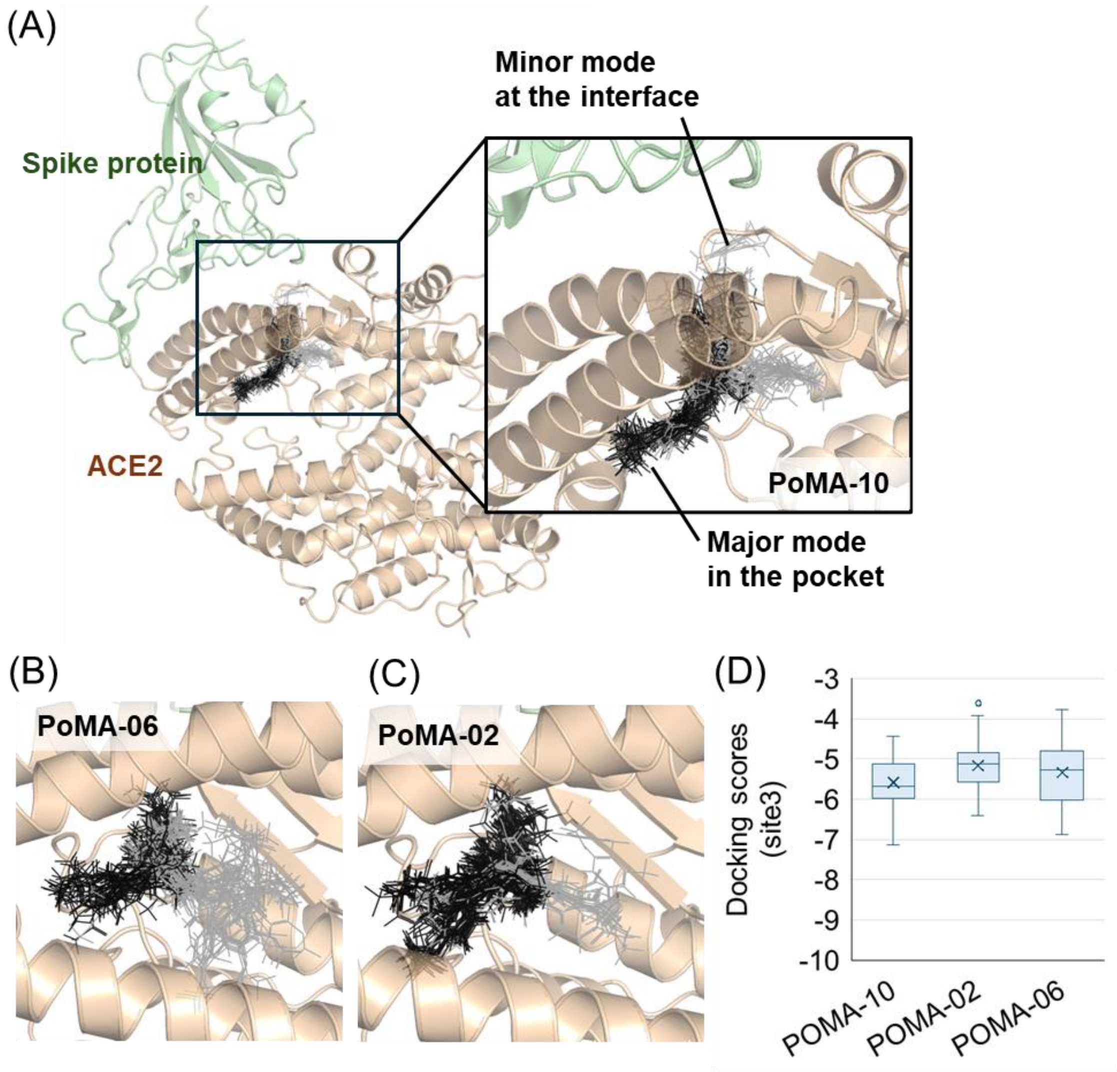

Figure S3. **Docked conformations of PoMA compounds at allosteric site 3 of ACE2.** (**A**–**C**) Overlaid multiple docking modes of PoMA-10, PoMA-06, and PoMA-02, respectively. Docking simulations indicate that PoMA-10 displays consistent and reproducible binding poses primarily at Site 3, located near the ACE2–spike interface, while PoMA-06 and PoMA-02 show more variable and less stable interactions. Major binding conformations are shown as black sticks, with minor poses represented in gray. (D) Distribution of docking scores (binding affinities in kcal/mol) for each compound at Site 3. PoMA-10 demonstrates slightly more favorable (i.e., more negative) scores compared to PoMA-06 and PoMA-02, supporting its stronger and more reliable binding at this site.

Figure S4. Enthalpy Energy (protein–protein interaction between ACE2 and Spike)

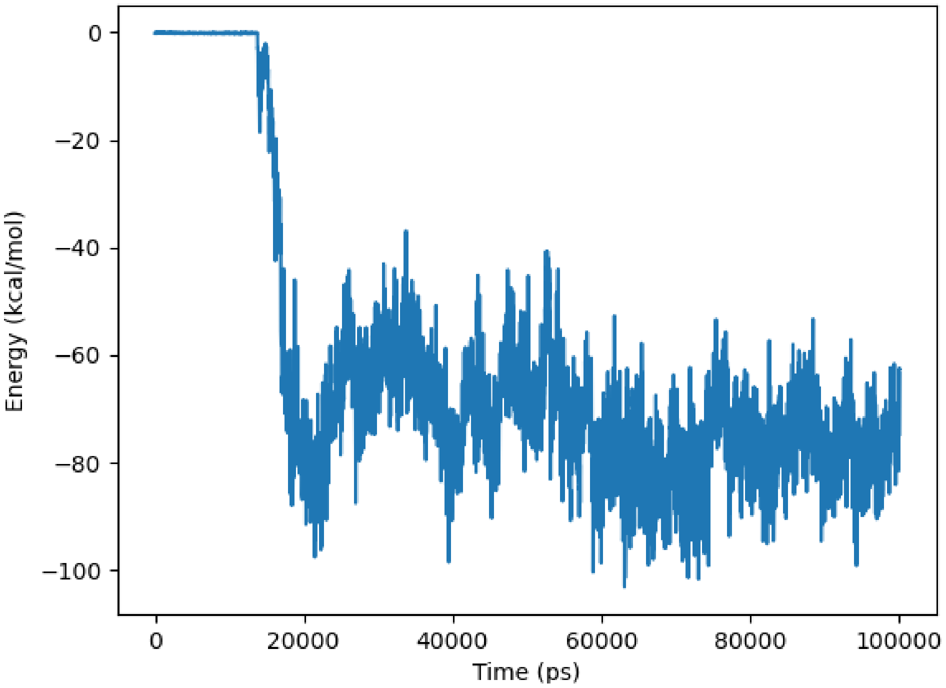

Figure S4. Time evolution of the protein–protein interaction energy between ACE2 and Spike over 100 ns of molecular dynamics. The interaction energy starts near zero, then drops sharply around 10–20 ns and thereafter fluctuates around a more negative plateau, indicating spontaneous approach, interface formation, and stabilization of the ACE2–Spike complex (more negative values reflect more favorable interactions).

Figure S5. Enthalpy Energy (Interaction between ACE2 and PoMA-10).

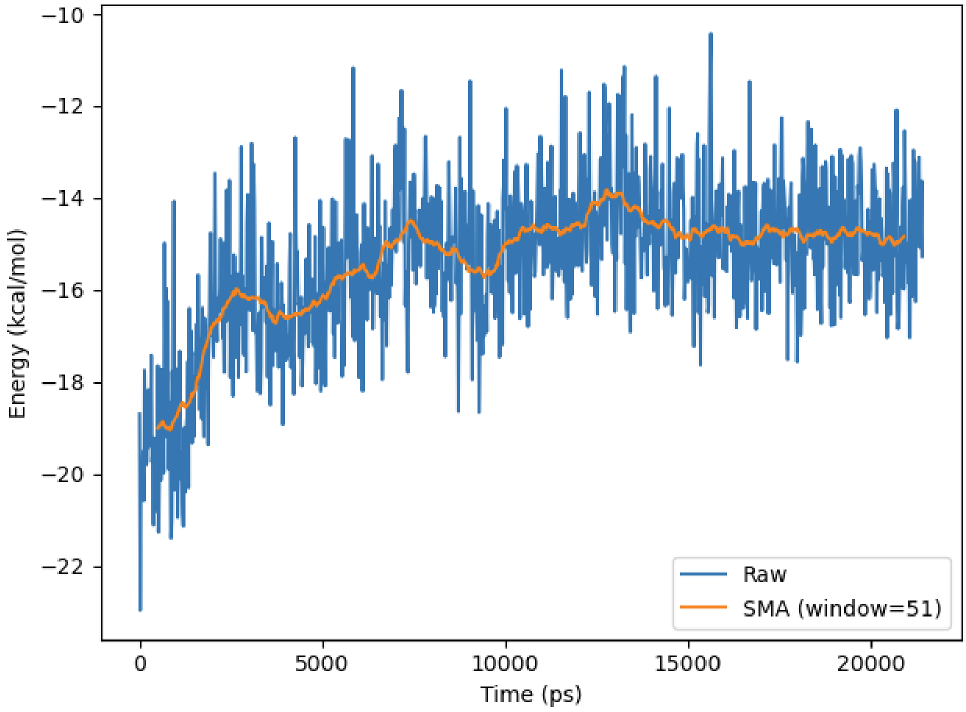

Figure S5. Time course of the ACE2–PoMA-10 interaction energy during a 20 ns simulation. Even as ACE2 separates from Spike protein, the ACE2–PoMA-10 interaction energy exhibits a trend toward stabilization.

Figure S6. Enthalpy Energy (protein-protein interaction between ACE2–PoMA-10 complex and Spike).

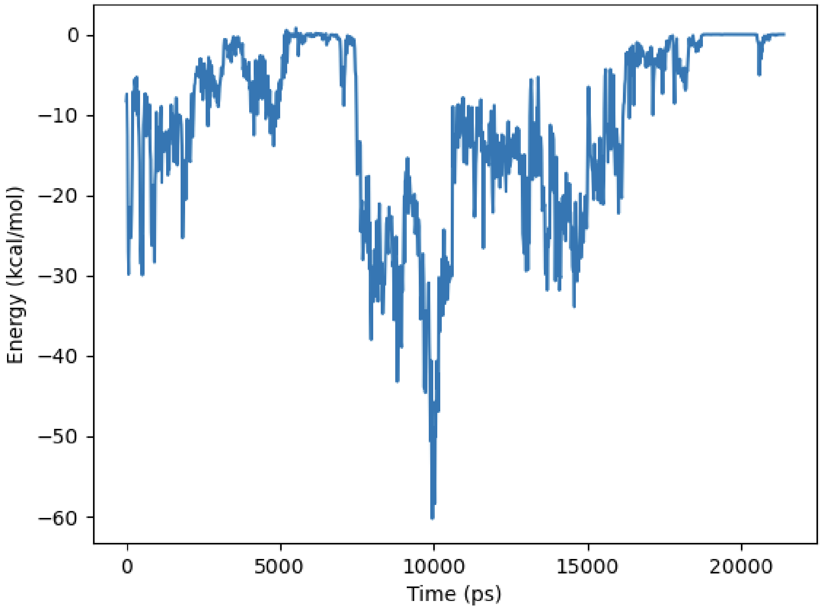

Figure S6. Time course of the protein-protein interaction energy between ACE2 and Spike during a 20-ns simulation in the presence of PoMA-10 (PoMA-10 remains bound to ACE2; see Fig. S5). The interaction energy between the ACE2–PoMA-10 complex and Spike is highly unstable for the first ~10 ns, with deep transient energy minima, then rises toward ~0 kcal·mol^{-1} for the remainder of the simulation. This behavior indicates progressive loss of contacts between the ACE2– PoMA-10 complex and Spike and effective disengagement of Spike. More negative values indicate more favorable interactions.

Figure S7. Toxicology analysis in mice following oral administration of PoMA-10.

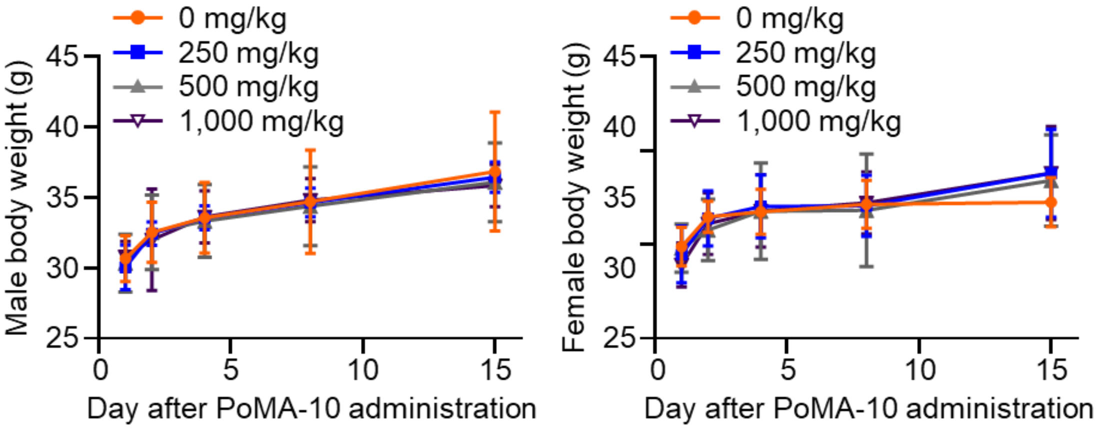

Figure S7. Effects of PoMA-10 on body weight following oral administration in mice. The selected doses of PoMA-10 (250, 500, and 1,000 mg/kg) were administered in either male or female mice. No abnormal signs were observed up to a dose of 1,000 mg/kg complex. There were no significant changes in body weight or mortality in either male or female mice.

Supplementary Videos 1 and 2

The molecular dynamics simulation results were divided into two main cases. In both simulations, the ACE2 and SARS-CoV-2 Spike proteins were initially placed 5 Å apart before the start of the production run.

Supplementary Video 1 is provided as a separate movie file.

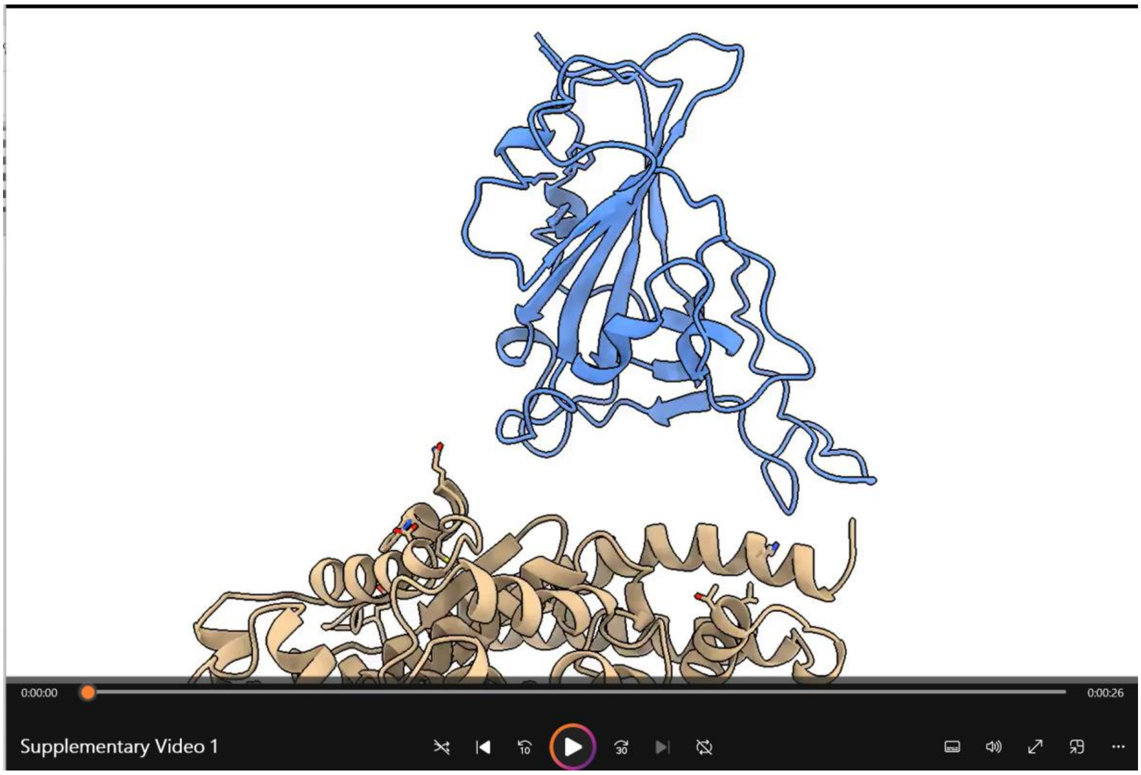

Supplementary Video 1. Case 1: Binding between ACE2 and the SARS-CoV-2 Spike protein.
This simulation reproduced the natural binding process of the SARS-CoV-2 Spike protein to ACE2 through molecular dynamics. As the Spike protein drew nearer to ACE2, the number of contact residues and the interaction energy gradually increased (Figure S4). The attached trajectory video (Supplementary Video 1) clearly shows the two proteins progressively approaching each other, with the center-of-mass (COM) distance decreasing from ~5.4 nm to 4.88 nm—a total closure of 5.2 Å. Because the initial configuration was not defined with a large separation, the numerical differences in distance are modest, yet they clearly show a consistent convergence of the binding interface toward a stable association.

Supplementary Video 2 is provided as a separate movie file.

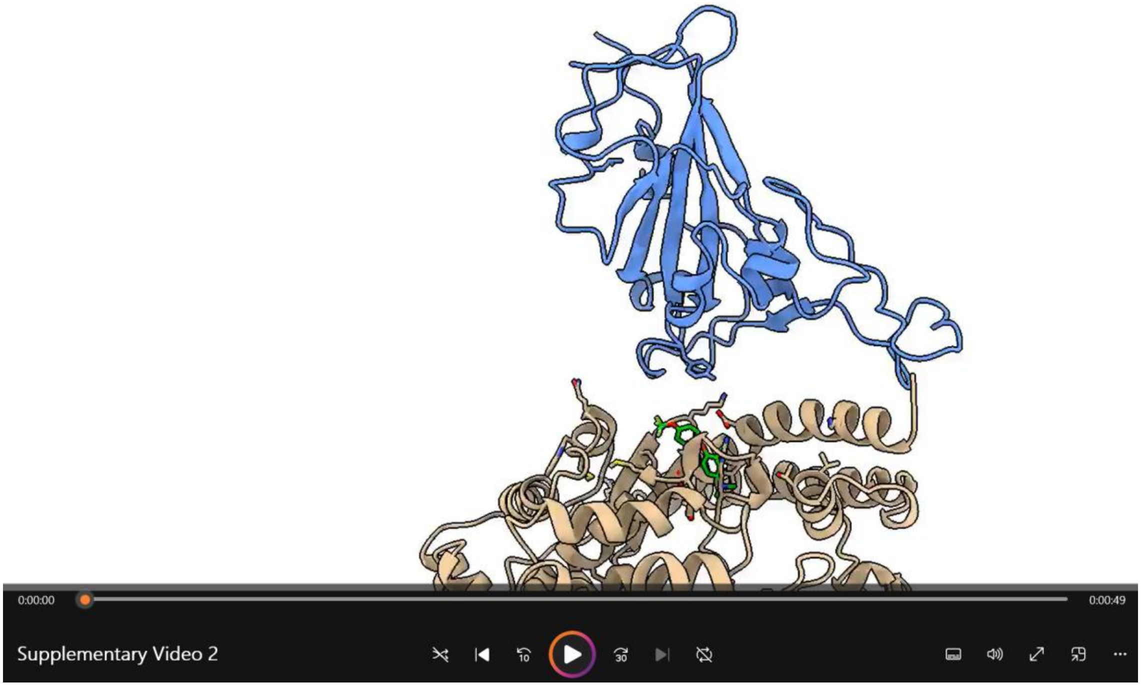

Supplementary Video 2. Case 2: Simulation of the ACE2–PoMA-10 complex.
In this case, the SARS-CoV-2 Spike protein failed to approach ACE2 due to the PoMA-10 bound to the ACE2 contact site. The ACE2–PoMA-10 complex was simulated without any external biasing or positional restraints, as in Case 1. During a 20 ns production run, the PoMA-10 remained stably bound to ACE2 and did not dissociate from the protein (Supplementary Video 2). The simulation revealed that the binding affinity between the SARS-CoV-2 Spike protein and ACE2 gradually weakened over time. Although the initial protein–PoMA-10 interaction energy slightly decreased, this reflected the stabilization of the ACE2–PoMA-10 complex as it converged toward equilibrium (Figure S5). Meanwhile, the protein–protein interface between ACE2 and the Spike protein exhibited significant fluctuations early in the trajectory and then gradually decayed toward zero, indicating loss of effective binding (Figure S6).

References

[1] Q. Wang, Y. Zhang, L. Wu, S. Niu, C. Song, Z. Zhang, G. Lu, C. Qiao, Y. Hu, K.Y. Yuen, Q. Wang, H. Zhou, J. Yan, J. Qi, Structural and Functional Basis of SARS-CoV-2 Entry by Using Human ACE2, Cell, 181 (2020) 894–904 e899.

[2] K. Dutta, Allosteric Site of ACE-2 as a Drug Target for COVID-19, ACS Pharmacol Transl Sci, 5 (2022) 179–182.

[3] A.W. Sousa da Silva, W.F. Vranken, ACPYPE - AnteChamber PYthon Parser interfacE, BMC Res Notes, 5 (2012) 367.

[4] X. He, V.H. Man, W. Yang, T.S. Lee, J. Wang, A fast and high-quality charge model for the next generation general AMBER force field, J Chem Phys, 153 (2020) 114502.

[5] P. Li, B.P. Roberts, D.K. Chakravorty, K.M. Merz, Jr., Rational Design of Particle Mesh Ewald Compatible Lennard-Jones Parameters for +2 Metal Cations in Explicit Solvent, J Chem Theory Comput, 9 (2013) 2733–2748.

[6] S. Jo, T. Kim, V.G. Iyer, W. Im, CHARMM-GUI: a web-based graphical user interface for CHARMM, J Comput Chem, 29 (2008) 1859–1865.
